# Regulation, structure, and activity of the *Pseudomonas aeruginosa* MexXY efflux system

**DOI:** 10.1128/aac.01825-24

**Published:** 2025-04-07

**Authors:** Logan G. Kavanaugh, Megan E. Hinrichsen, Christine M. Dunham, Graeme L. Conn

**Affiliations:** 1 Department of Biochemistry, Emory University School of Medicine 12239, Atlanta, Georgia, USA; 2 Graduate Program in Microbiology and Molecular Genetics, Emory University 1371, Atlanta, Georgia, USA; 3 Department of Chemistry, Emory University 221304, Atlanta, Georgia, USA; 4 Graduate Program in Biochemistry, Cell and Developmental Biology, Emory University 1371, Atlanta, Georgia, USA; 5 Emory Antibiotic Resistance Center, Emory University School of Medicine 12239, Atlanta, Georgia, USA; Houston Methodist Hospital and Weill Cornell Medical College, Houston, Texas, USA

**Keywords:** *Pseudomonas*, efflux, resistance-nodulation-division (RND), antibiotic resistance

## Abstract

The current crisis in bacterial antibiotic resistance can be attributed to the overuse (or misuse) of these essential medicines in healthcare and agriculture, coupled with the slowed progression of new drug development. In the versatile, opportunistic pathogen *Pseudomonas aeruginosa*, the Resistance-Nodulation-Division (RND) efflux pump MexXY plays critical roles in both cell physiology and the acquisition of multidrug resistance. The *mexXY* operon is not constitutively expressed, but this process is instead controlled by a complex network of multiple interconnected regulatory mechanisms. These include induction by several of the pump’s ribosome-targeting antibiotic substrates and transcriptional repression and anti-repression processes that are themselves influenced by various cellular factors, processes, or stresses. Although extensive studies of the MexXY complex are currently lacking as compared to other RND efflux pumps such as *Escherichia coli* AcrAB-TolC, recent studies have provided valuable insights into the MexXY architecture and substrate profiles, including its contribution to clinical resistance. Furthermore, while MexXY primarily associates with the outer membrane protein OprM, emerging evidence suggests that this transporter-periplasmic adaptor pair may also partner with other outer membrane proteins, potentially to alter the efflux substrate profile and activity under specific environmental conditions. In this minireview, we summarize current understanding of MexXY regulation, structure, and substrate selectivity within the context of clinical resistance and as a framework for future efflux pump inhibitor development.

## INTRODUCTION

Bacteria have developed or acquired resistance to every antibiotic currently in use. This crisis in antibiotic resistance can be attributed to their overuse in healthcare, a problem compounded by the coronavirus disease 2019 (COVID-19) pandemic (1), and extensive use in agriculture, as well as the slowed progress of new drug discovery and development (2). Bacteria evolve or acquire resistance to antibiotics through four main mechanisms: limiting drug uptake, drug inactivation, target alteration via mutation or chemical modification, and active drug export via efflux. Efflux pumps are found across all prokaryotes and can transport a diverse range of substrates out of the cell including heavy metals, dyes, detergents, bile salts, and antibiotics.

Of the five major efflux pump families in Gram-negative pathogens (3), the Resistance-Nodulation-Division (RND) efflux pumps play a central role in intrinsic antibiotic resistance and virulence (4). RND efflux pumps are pervasive among difficult-to-treat Gram-negative pathogens and thus a prime target for the development of efflux pump inhibitors (EPIs) that could be used as antibiotic adjuvants. Although challenging, this approach has the advantages of rejuvenating already approved antibiotics, circumventing the need to identify novel antibiotic targets and drugs, and targeting a mechanism that confers resistance to a broad range of antibiotics. Of the 12 current bacterial threats designated as urgent or serious by the United States Centers for Disease Control and Prevention (5), eight contain at least one RND efflux pump, including *Acinetobacter baumannii, Neisseria gonorrhoeae, Escherichia coli, Klebsiella pneumoniae,* and *Pseudomonas aeruginosa*. Studies are therefore urgently needed to determine the molecular bases of regulation, structure, and activity of RND efflux systems as these processes remain poorly characterized in many pathogens outside of a small number of notable exceptions (6–8).

*P. aeruginosa* presents an excellent system for studying RND efflux function and substrate recognition due to the high prevalence of RND efflux pumps, the robust ability to perform *in vivo* genetic experiments, and the large collection of available whole genome sequences of clinical isolates. In *P. aeruginosa* PAO1, there are at least 12 RND efflux pumps, of which four contribute significantly to antibiotic resistance: MexAB-OprM, MexCD-OprJ, MexEF-OprN, and MexXY-OprM (9). These four pumps have overlapping but distinct antibiotic substrate profiles: e.g., MexAB-OprM can export β-lactams but not aminoglycosides, MexXY-OprM can export aminoglycosides but not most β-lactams, and both can export macrolides, tetracyclines, and chloramphenicol (10, 11). Additionally, each pump is independently regulated, with some pumps being constitutively “on” (e.g., MexAB-OprM) and others “off” under normal conditions (e.g., MexXY) (12, 13). Understanding the role of efflux-mediated antibiotic resistance in *P. aeruginosa* is also of clinical importance due to this pathogen’s contribution to 32,600 infections, 2,700 deaths, and $767 million in healthcare costs in the United States alone (5) and >300,000 deaths worldwide in 2019 (14). More recent updates suggest this situation has continued to worsen, with *P. aeruginosa* linked to ~10% of deaths related to antimicrobial-resistant infections and the dissemination of multidrug-resistant *P. aeruginosa* in US hospitals increasing by 32% during the COVID-19 pandemic in 2020 (1, 15). *P. aeruginosa* can colonize multiple areas of the body including the eye, wound, and lung and can cause both acute nosocomial infections, such as pneumonia, sepsis, and urinary tract infections, as well as chronic infections, e.g., in individuals with cystic fibrosis (CF) (5). For pediatric patients with CF, aminoglycosides are a critical component of antipseudomonal therapy for the treatment of lung infections (9), but treating such infections is becoming challenging due to intrinsic resistance conferred by MexXY-OprM (16, 17). In this mini-review, we describe the field’s current understanding of MexXY regulation, structure, and substrate selectivity within the context of clinical resistance and as a framework for future EPI development efforts.

## *MexXY* GENE EXPRESSION IS REGULATED BY MULTIPLE MECHANISMS

**Fig 1:**
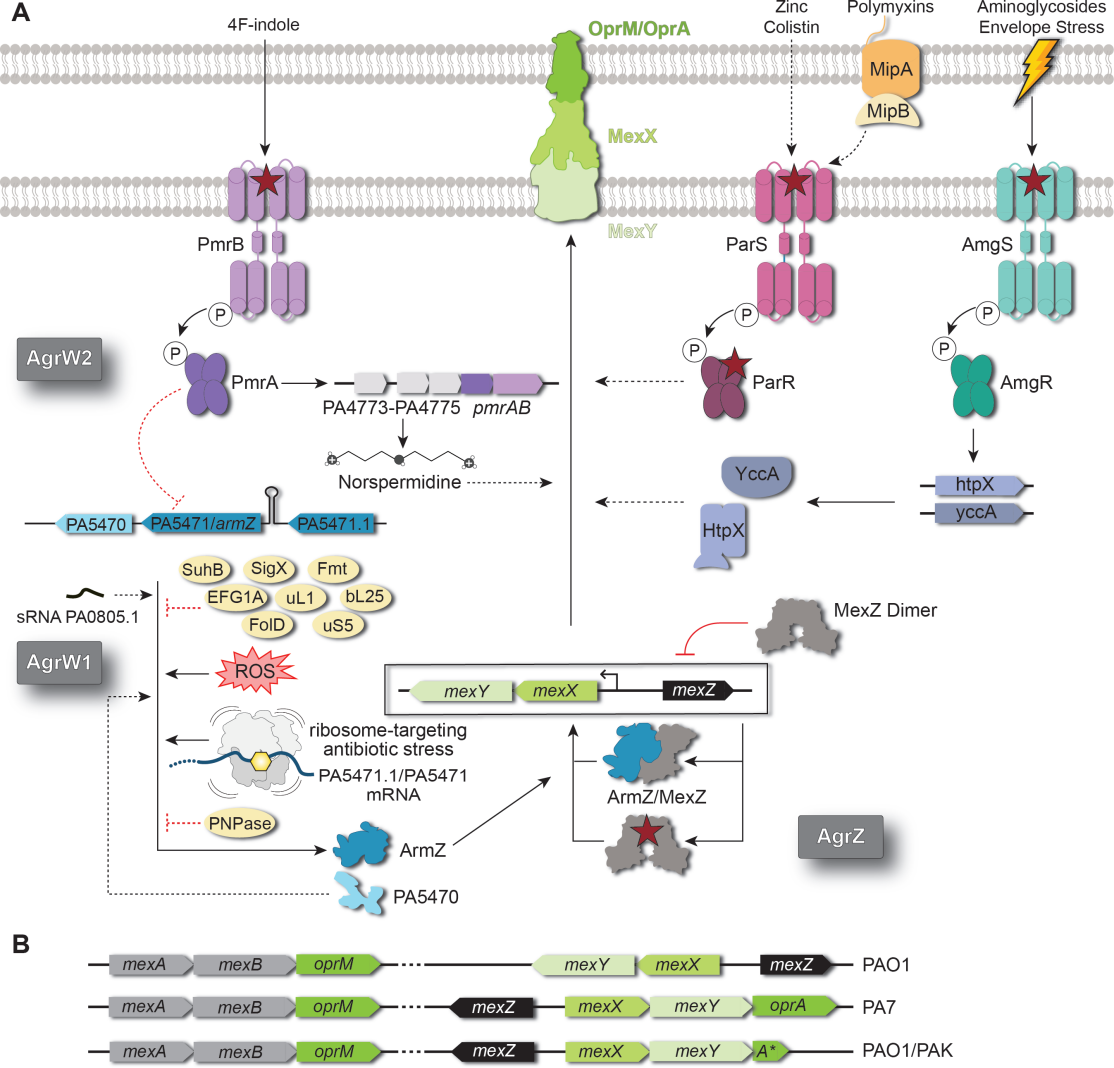
Genetic organization and regulation of *mexXY* expression. (A) The *mexXY* operon is transcriptionally repressed by the MexZ dimer (gray). MexZ repression can be inhibited through ArmZ (blue) which is regulated through various proteins (SuhB, SigX, Fmt, elongation factor G1A, bL25, uL1, uS5, FolD; yellow ovals), sRNA (PA0805.1), and physiological conditions, including presence of ribosome-targeting antibiotics or reactive oxygen species (ROS). ArmZ is also posttranscriptionally regulated by PNPase protein (yellow oval). ArmZ-independent mechanisms can also regulate *mexXY* through TCS with different stimuli (AmgRS, ParRS, and PmrAB). Upregulation of *mexXY* can occur via mutations (denoted with red stars) in MexZ (*AgrZ* strains), ArmZ (*AgrW1* strains), or in TCS components (*AgrW2* strains). Black solid arrows represent processes of transcription/translation or direct interactions known to regulate protein expression; black dashed lines represent potential additional, currently undefined mechanisms that result in expression of the MexXY or ArmZ proteins. Red solid and dashed lines with bars are the same but for mechanisms of gene repression. For the TCS systems, the “P” in the circle represents the phosphate group, and the curved black lines indicate the movement of the phosphate through the TCS kinase activity to the response protein. (B) Organization of the *mexAB* and *mexXY* operons within different *P. aeruginosa* strains: PAO1, PA7, and some PAO1/PAK strains which have retained a fragment of the OprA OMP (A*). Arrows indicate the directionality of transcription for each gene encoded.

As *mexXY* is not constitutively expressed and its upregulation is a major contributor to clinical aminoglycoside resistance, early efforts to identify potential regulators of the *mexXY* operon examined the genomes of pan-aminoglycoside-resistant isolates (18–20). These studies identified MexZ as a repressor of *mexXY,* and the attributable changes in *mexXY* expression were categorized into two distinct classes with either inactive MexZ (aminoglycoside resistance due to MexZ or AgrZ strains) or active MexZ (aminoglycoside resistance due to unknown or AgrW strains) (18–20) (Fig. 1A). Subsequently, the origin of the observed aminoglycoside resistance in AgrW strains was identified through whole-genome sequencing as either mutations acquired in the anti-repressor of MexZ (ArmZ; resulting in AgrW strain subtype AgrW1), or in two-component systems (TCS) at the outer membrane (resulting in AgrW strain subtype AgrW2) (18). Additionally, *mexXY* expression was found to be upregulated in the presence of ribosome-targeting antibiotics, including tetracycline, erythromycin, chloramphenicol, and gentamicin, all of which are efflux substrates of MexXY-OprM (21, 22). The current understanding of the activators and repressors of *mexXY* and how they relate to drug-induced expression, as discussed further in the following sections, is summarized in Fig. 1A and Table 1.

**TABLE 1:** Summary of regulatory factors controlling *mexXY* expression^*a*^

MexXY regulator	Function	Expression	Regulation/mechanism	MexXY expression
*mexZ*	MexZ: transcriptional repressor	Constitutively active	Transcriptional repressor	Increased—when derepressed (ArmZ) or mutated
*armZ*	ArmZ: RtcB-like RNA ligase; anti-repressor of MexZ	Various (see below)	Derepresses the MexZ repressor	Increased—when ArmZ expressed
*parRS*	ParRS TCS: recognizes cationic peptides (polymyxin B, colistin)	Constitutively active	Unknown	Increased—induced by colistin or mutation
*mipBA*	MipBA: membrane-embedded polymyxin B co-sensors	Constitutively active	Unknown; potentially induces ParRS	Increased—induced by polymyxin B
*pmrAB*	PmrAB TCS: recognizes cations and lipopolysaccharide	Constitutively active	Unknown; potentially through PA4773-PA4775	Increased—upon mutation
*amgRS*	AmgRS TCS: controls damage due to depolarization of the bacterial membrane	Constitutively active	Unknown; potentially via *htpX* and *yccA*	Increased—when induced/mutated
PA4773-PA4775	Norspermidine synthesis operon	*pmrB* mutation (Q105P)	Unknown	Increased
*htpX*	HtpX: zinc-dependent endoprotease in the IM; important for Sec-mediated protein translocation	*amgS* mutation; aminoglycoside exposure	Unknown	Increased
*yccA*	YccA: modulator of FtsH protease; important for Sec-mediated protein translocation	*amgS* mutation; aminoglycoside exposure	Unknown	Increased
Zinc	Multiple functions	N/A	Potentially through induction of ParRS	Increased

ArmZ Regulator	Function	Expression	Regulation/mechanism	ArmZ expression
PA5471.1	Leader peptide; important for regulation of PA5470/*armZ*	Constitutively active	Transcriptional attenuation with impaired/stalled ribosomes	Increased—under specific conditions (e.g., ribosome stalling)
*pnp*	Polynucleotide phosphorylase (exonuclease); rRNA degradation, tRNA processing, and gene regulation	Constitutively active	Regulates ArmZ translation; mechanism unknown	Decreased
*suhB*	SuhB: inositol monophosphate; associated with rRNA biogenesis	Constitutively active	Regulates transcription and translation via interactions with the ribosome; specific mechanism unknown	Decreased
*folD*	FolD: bi-functional methylenetetrahydrofolate dehydrogenase/cyclohydrolase	Constitutively active	Unknown	Decreased
*fmt*	Fmt: tRNA^fMet^-formyl transferase	Constitutively active	Unknown	Decreased
ROS	N/A	N/A	Unknown; potentially through ribosome disruption	Increased
*sigX*	σ-factor *S*igX; cytoplasmic regulator	Constitutively active	Unknown; influences ArmZ regulation	Decreased
*rplA*, *rplY, rspE,* *rplU*/*rpmA*	Ribosomal proteins (uL1, bL25, uS5, bL21/bL27)	Constitutively active	Unknown	Decreased
*fusA1*	Elongation factor G1A (EF-G1A): aids in mRNA-tRNA translocation	Constitutively active	Unknown	Decreased
*pmrAB*	PmrAB TCS: recognizes cations and LPS	Constitutively active	Unknown	Decreased—when induced with 4F-indole

^*a*^ N/A, Not applicable.

### Direct regulation of mexXY expression: MexZ and its anti-repressor ArmZ

#### *The* mexXY *repressor MexZ*

**Fig 2:**
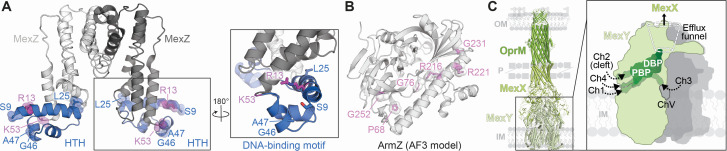
Structural models of MexZ , ArmZ and MexXY-OprM. (A) Structure of the MexZ dimer (PDB: 2WUI) with DNA-binding domain residues within the HTH highlighted in each protomer in blue: S9, L25, G46, and A47. Residues predicted to interact with ArmZ are highlighted in purple: R13 and K53. (B) AlphaFold3 model of ArmZ (PDB: AFQ9HT99F1) with residues predicted to interact with MexZ highlighted in light purple: P68, G76, R216, R221, G231, and G252. (C) Homology model of the MexXY-OprM efflux system and schematic representation of key features of MexY (boxed) discussed in the main text. Outer membrane, inner membrane, and periplasm are depicted and denoted OM, IM, and P, respectively.

The *mexXY* operon is primarily regulated via the transcriptional repressor MexZ, encoded by a third open-reading frame 263 base pairs (bp) upstream of *mexX* (23) (Fig. 1B). *mexZ* is transcribed divergently from *mexX,* and the encoded protein MexZ is a member of the TetR family of transcriptional repressors, homologous to those associated with other RND efflux systems, e.g., AmrR in *Burkholderia pseudomallei*, MtrR in *N. gonorrhoeae*, and AcrR in *E. coli* (23–25). The crystal structure of MexZ confirmed that it adopts a canonical dimeric TetR repressor structure and binds at the −104 to −66 region upstream of *mexX* containing the *mexXY* promoter region. Similar to MtrR, MexZ binds to a 20 bp inverted repeat sequence, resulting in decreased transcription of *mexXY* (23, 26–28). MexZ contains an N-terminal helix-turn-helix (HTH) motif that binds and represses at the *mexXY* operator DNA and a C-terminal ligand binding domain of unknown function (23, 27) (Fig. 2A).

Clinical isolates with inactivated MexZ repressor (i.e., AgrZ) are the most common class of aminoglycoside-resistant clinical strains (18, 20), with resistance being twofold to eightfold greater than for strains with a functional repressor (19) (Fig. 1A, bottom right). In isolates recovered from CF patients with chronic *P. aeruginosa* infections, amino acid substitutions are found within both the MexZ HTH motif (S9F, L25P, G46V, and A47P; Fig. 2A) and the orphan C-terminal ligand binding domain (P151L, L154P, S202F, and L205P) (27, 29). Electrophoretic mobility shift assay analyses of variant MexZ protein binding to operator DNA containing the *mexXY* promoter revealed that HTH motif substitutions decreased binding, with G46V completely abolishing the interaction and S9F and L25P reducing affinity by 100-fold (27). In contrast, C-terminal substitutions (S202F and P151L) had little to no effect, indicating that they impact aminoglycoside resistance via a distinct mechanism that remains to be determined. Since *mexXY* expression is upregulated (22, 30) and TetR repressors contain a ligand binding domain, initial speculation was that substrate antibiotics might bind MexZ resulting in derepression of *mexXY* (26). However, the presence of antibiotics had no effect on the MexZ-DNA interaction (26), suggesting that there are alternative pathways to control *mexXY* expression.

#### ArmZ—the MexZ anti-repressor

Pan-aminoglycoside-resistant *P. aeruginosa* clinical isolates with overexpression of the *mexXY* operon were also identified that possessed an intact MexZ repressor (i.e., AgrW isolates), suggesting an alternative regulatory pathway. The first class of AgrW1 isolates were discovered using transposon insertion mutagenesis that identified additional genes responsible for loss of aminoglycoside resistance (30). PA5471 (later named ArmZ) was found to be necessary for aminoglycoside resistance in a MexXY- and MexZ-dependent manner. This observation suggests that ArmZ derepresses the *mexXY* operon either through direct interaction with MexZ or via an alternative pathway leading to decreased MexZ action (30) (Fig. 1A*,* bottom left).

ArmZ and MexZ were shown to interact directly via a two-hybrid assay using an *E. coli* harboring a chromosomal *lacZ* gene with a hybrid *lexA* operator sequence in its promoter region, and ArmZ and MexZ each fused to LexA lacking its dimerization domain (31). Only when ArmZ and MexZ were coexpressed was β-galactosidase activity reduced, indicating that ArmZ directly interacts with MexZ (31). Subsequent studies using a different two-hybrid system suggested that truncation or substitution at the MexZ HTH motif (K53E and R13H; Fig. 2A) abolished or diminished interactions (32). A collection of ArmZ residues within a large C-terminal α-helix whose substitution resulted in decreased interaction between ArmZ and MexZ also provided evidence for their interaction (Fig. 2B). The ArmZ C-terminal α-helix is now recognized as the MexZ interacting domain, and its interaction with the N-terminal MexZ HTH motif blocks repressor binding at the *mexXY* DNA operator (28, 31, 32).

Upregulation of both *mexXY* and *armZ* occurs in the presence of ribosome-targeting antibiotics, including aminoglycosides and chloramphenicol (22, 30). However, the connection between the ribosome, ribosome-targeting antibiotics, and regulation of *armZ* was unclear until the discovery of the leader peptide PA5471.1, which regulates *armZ* and thus *mexXY* expression (33). Thus far, it is proposed that the leader peptide PA5471.1 regulates expression via a transcriptional attenuation mechanism, but additional studies are needed to confirm this. *armZ* regulation was also hypothesized to be independent from *trans*-translation, the bacteria-specific process for rescuing stalled ribosomes (30). However, it is now known that alternative rescue pathways exist which were not explored in this study. Therefore, a connection between the regulation of *mexXY* expression and *trans*-translation cannot yet be conclusively ruled out.

#### armZ *regulation*

Multiple pathways for *armZ* regulation have been identified, including via the leader peptide PA5471.1, regulatory proteins, and through various other cellular processes (34–38) (Table 1). PA5471.1 is constitutively expressed under normal *P. aeruginosa* growth conditions and predicted downstream transcriptional terminators in the intergenic region have been hypothesized to prevent expression of PA5471/*armZ* (33). However, ribosome-targeting antibiotics somehow influence translation of PA5471.1 in a manner that does not change the levels of the PA5471.1 leader peptide but, instead, causes a restructuring of downstream mRNA to prevent transcriptional termination and allows downstream expression of PA5471/ArmZ (33). Upregulation of ArmZ in the absence of ribosome-targeting antibiotics was identified in some pan-aminoglycoside resistant isolates containing a seven-nucleotide leader peptide deletion (PA5471.1∆16-22) which results in transcriptional readthrough and constitutive expression of *armZ*, ultimately leading to expression of MexXY (18).

Two additional proteins are involved in *armZ* regulation, a polynucleotide phosphorylase (PNPase) (34) and the RNA polymerase- and ribosome-associated SuhB (35, 39, 40). PNPase is a highly conserved exonuclease which has been implicated in rRNA degradation, tRNA processing, and small RNA (sRNA) gene regulation (41–45). Expression of an inactive PNPase lacking the RNA-binding domains (Pnp∆KH-S1) in PAO1 increased the stability of two sRNAs, RsmY and RsmZ, increased expression of the H1 type VI secretion system, and increased resistance to ciprofloxacin and aminoglycoside antibiotics (34, 46, 47). On first exposure to aminoglycosides, PAO1 Pnp∆KH-S1 had a similar MIC to the wild type, but upon second exposure exhibited up to 10,000-fold higher survival rates. This dramatically increased tolerance appeared to be a consequence of MexXY overexpression via inhibition of posttranscriptional regulation of the 5′-untranslated region of *armZ* mRNA (34). While the exact role of PNPase is unclear under normal conditions, the authors hypothesized that PNPase degrades sRNAs that bind to the 5′-untranslated region of *armZ* preventing its translation. In contrast to sRNA regulation of *armZ* via PNPase, the recently observed overexpression of the sRNA PA0805.1, which increases expression of *mexXY* (48), was found to be PNPase-independent (34).

The conserved inositol monophosphatase protein *E. coli* SuhB is associated with elongating RNA polymerase in a NusB-dependent manner during ribosomal RNA transcription (40, 49, 50). Through immunoprecipitation and mass spectrometry analysis, *P. aeruginosa* SuhB was shown to directly associate with the ribosome (35). Deletion of *P. aeruginosa suhB* resulted in increased *armZ* and *mexXY* transcript levels*,* which were predicted to be through increased ribosome stalling on the PA5471.1 leader peptide (35). However, defects in *P. aeruginosa suhB* are involved in a wide range of processes including the downregulation of type III secretion systems, upregulation of GacA (a positive regulator of virulence factors), sRNAs RsmY and RsmZ, and type IV secretion systems (51). Mutations in *suhB* were also associated with increased sensitivity to β-lactams, but increased resistance to aminoglycosides (35, 52). Thus, despite evidence that *E. coli* SuhB regulates rRNA transcription and ribosome biogenesis (40, 49, 50), its specific role in the regulation of *armZ* in *P. aeruginosa* remains to be determined.

Other pathways that can also influence *mexXY* expression via ArmZ upregulation include protein formylation, generation of reactive oxygen species (ROS), or cytoplasmic regulators. LBM415, a peptide deformylase inhibitor, showed promising results as an antibiotic with activity against Gram-negative bacteria (36). However, LBM415 also increased the mutation frequency of genes encoding enzymes important for formylation of initiator methionine tRNA, such as bi-functional methylenetetrahydrofolate dehydrogenase/cyclohydrolase (*folD*) and tRNA^fMet^-formyl transferase (*fmt*) (36). These mutations result in the elimination or reduction in formylmethionine (“Fmt bypass”), which overall decreases translation and results in constitutive expression of *mexXY* via ArmZ upregulation (36). However, the details of this pathway and how it connects to *armZ* expression are still unknown. Additionally, ROS exposure was found to induce expression of *armZ* and *mexXY,* which may be due to disruption of the ribosome resulting from RNA chemical modifications (37, 53). ROS production from chronic inflammation in the lungs of individuals with CF can also result in MexXY upregulation even before exposure to antibiotic treatment (37). Deletion of the cytoplasmic regulator σ-factor *sigX* caused significantly increased expression of *mexXY* and *armZ* (up to 13- and 7-fold, respectively), in addition to genes associated with motility, twitching, adhesion, and stress response (38). Lastly, a predicted peptide chain release factor (PA5470/*prfH*), located adjacent to *armZ* and previously shown to alleviate macrolide-induced ribosome stalling, was also upregulated through the deletion of *sigX* (38, 54). For each of these pathways, the precise connections to *mexXY* overexpression have yet to be elucidated, and it is unclear under which physiological conditions these distinct pathway(s) contribute to pump overexpression. Addressing these gaps represents an important next step in fully defining the regulation of *mexXY* expression and its contribution to cell physiology.

#### Disruption to the ribosome

A second class of AgrW1 variant strains contained disruptions in genes encoding ribosomal proteins *rplA* (uL1), *rplY* (bL25), or *rspE* (uS5), within the promoter region of *rplU-rpmA* which encodes ribosomal proteins bL21 and bL27, or in *fusA1*, encoding elongation factor G1A which translocates mRNA-tRNA during translation elongation (22, 55–59) (Table 1; Fig. 1A; bottom left). Of note, *fusA1* is commonly mutated in *P. aeruginosa* strains isolated from individuals with CF (57). While these mutations were correlated with increased aminoglycoside resistance and *mexXY* expression, the mechanism by which this occurs is currently unknown. However, as these disruptions to ribosomal proteins or factors may inhibit ribosome activity, stalling at PA5471.1 may be connected to the impact of these mutations.

### Induction of MexXY via signaling at the membrane

#### Two-component systems

The second class of pan-aminoglycoside resistant isolates possess an intact *mexZ* gene (AgrW2) but overexpress *mexXY* due to mutations in genes encoding TCS, including ParRS, PmrAB, and AmgRS (18, 60–65) (Fig. 1A, top). ParRS recognizes external stimuli such as cationic peptides and is essential in bacterial resistance to polycationic peptide antibiotics, e.g., polymyxin B, colistin, and indolicidin (18, 60, 61). Strains with amino acid substitutions within either ParR (e.g., M59I or E156K) or ParS (e.g., L14Q, A138T, or L137P) cause overexpression of *mexXY* (18, 60, 61), but induction occurs via an ArmZ-independent mechanism as deleting *armZ* had no effect on *mexXY* upregulation by colistin (60, 61). Even though aminoglycosides are polycationic compounds, ParRS does not appear to be involved in aminoglycoside-mediated regulation of *mexXY* as induction of *mexXY* was maintained in a ∆*parRS* strain treated with gentamicin (60). A recent study has also revealed the involvement of a ParRS-regulated operon encoding *mipBA*, as a co-sensor to polymyxin B, to induce MexXY expression (66).

MexXY is upregulated in a strain harboring a Q105P substitution in *pmrB*, which encodes part of PmrAB, a TCS that responds to cations and modulates outer membrane lipopolysaccharides (62). Aminoglycoside resistance was seen in PmrAB variant strains, and this effect was proposed to be due to the upregulation of PA4773-PA4775, a putative norspermidine synthesis operon (67). However, the mechanism by which norspermidine is involved in *mexXY* expression is still to be determined. Additionally, treatment of PAO1 with 4F-indole resulted in decreased *armZ* and *mexXY* expression while increasing *mexZ* expression, which was suggested to occur via PmrAB and led to an increased susceptibility to kanamycin (68).

Another TCS proposed to play an important role in the induction of *mexXY* expression is AmgRS, which controls damage from aminoglycoside exposure and the resulting depolarization of the bacterial cytoplasmic membrane due to mistranslated polypeptides (63). Substitutions in AmgS (e.g., V121G, R182C, or D106N) result in an increase in *mexXY* expression in an ArmZ-independent manner (63–65) and two genes regulated by AmgRS, *htpX,* and *yccA,* are required for AmgRS-dependent induction of *mexXY* by aminoglycosides (63, 69). HtpX is a zinc-dependent endoprotease in the *E. coli* inner membrane (IM) (70) and YccA is a modulator of the FtsH protease (71). Both proteins may play a role in Sec-mediated protein translocation. AmgRS-mediated induction of MexXY was compromised upon exposure to rifampicin in addition to aminoglycosides, which decreased expression of AmgRS target genes (specifically *htpX* and *yccA*) (69). Aminoglycosides with different chemical structures result in variable levels of *mexXY* expression, and such differences may influence which regulatory pathway(s) are involved (69). For example, 4,5-substituted deoxystreptamine containing aminoglycosides are better potentiators of AmgRS-regulated resistance compared to those with a 4,6-disubstituted ring organization (65, 69). While connections between TCS and increased *mexXY* expression have been observed, the pathways that are involved and how TCS disruption leads to eventual upregulation of *mexXY* need to be studied further to understand the complex regulation of this efflux pump (Table 1).

#### Divalent cations

Aminoglycosides are polycationic compounds that bind to the negative bacterial cell wall during entry. This process can be antagonized in the presence of divalent cations (e.g., Mg^2+^ and Ca^2+^) and cationic dipeptides (MC-207,110; phenylalanine arginyl β-napthylamide; PAβN) in a MexXY-dependent manner (72, 73). Additionally, the divalent cation, Zn^2+^ can induce *mexXY* in an AmgRS- and ArmZ-independent but potentially ParRS-dependent manner; this effect was found to be unique to Zn^2+^, as Mg^2+^ did not induce *mexXY* expression at the same concentrations (74). These findings suggest that an additional Zn^2+^-dependent regulator, potentially through ParRS, may be involved in MexXY regulation.

## *MexXY* STRUCTURE AND SUBSTRATE SELECTIVITY

### Canonical RND efflux pump structure

RND pumps are tripartite protein complexes composed of multimeric assemblies of an IM transporter protein (e.g., MexY), a periplasmic adaptor protein (PAP, e.g., MexX), and an outer membrane protein (OMP, e.g., OprM) (75–77) (Fig. 2C). RND transporters are IM-spanning homo-trimeric protein complexes that function as drug/H^+^ antiporters. Each protomer of the trimer contains 12 transmembrane (TM) helices (TM1–TM12) and a periplasmic domain comprising the pore (with PN1, PN2, PC1, and PC2 regions) and docking (with DN and DC) subdomains (78). The pore subdomain contains two binding pockets that are critical for substrate uptake and subsequent efflux, the proximal and distal binding pockets (PBP and DBP, respectively) (Fig. 2C). These internal cavities are separated by the functionally conserved glycine-rich “switch loop.” Studies of chimeric RND transporter proteins, e.g., engineered to contain the pore subdomain of one transporter and the TM subdomain of another, revealed the periplasmic pore and docking subdomains to be necessary and sufficient for substrate discrimination (76, 79–86), while the TM domain is necessary for proton relay driven by the proton motive force but is most likely not the primary location for substrate translocation (87).

The determination of high-resolution cryogenic electron microscopy (cryo-EM) structures of *E. coli* AcrAB-TolC and *P. aeruginosa* MexAB-OprM have suggested four potential entry channels (Ch1-4) for substrates from the periplasm (76, 86, 88, 89), in addition to highlighting functional dynamics of the RND transporter (Fig. 2C). Ch1, located between PC1 and TM8 of each protomer, and Ch2 (also known as the “cleft”) located between PC1 and PC2, both lead to the PBP. In contrast, Ch3, located within the vestibule channel (ChV) which is formed between PN2 of one protomer and PC2 of a second protomer, and Ch4, located at the TM1/TM2 grooves, both lead directly to the DBP. The importance of the vestibule and/or cleft channels as the primary periplasmic entry channels for antibiotics into the transporters AcrB and MexB was further corroborated with functional studies (e.g., mutational analysis and chimeric proteins) (75, 76, 78, 79, 89–94). However, neither of these transporters has been implicated in aminoglycoside efflux, which in *P. aeruginosa* is primarily accomplished via MexXY (10). The structure of one transporter, which can efflux aminoglycosides, *E. coli* AcrD, was determined and has implicated the negatively charged vestibule ceiling residues D99, D101, and E102, as being important for aminoglycoside recognition (95). This observation was further corroborated using alanine substitutions of each residue, which resulted in decreased cell growth in the presence of gentamicin as compared to wild type (95).

Structures of AcrB and MexB, in both their *apo* and substrate-bound states, show that each protomer can cycle through three main conformational states: loose (L), tight (T), and open (O) (92, 96, 97). The cycling of the LTO conformational states results in a peristaltic motion that moves substrate from the periplasmic entry channel into the PBP/DBP and subsequently the exit funnel leading to the PAP. Currently, no high-resolution structures of MexXY-OprM or its unique individual components (i.e., MexX and MexY) are available. However, sequence analyses and protein modeling suggest that the general structure, functional dynamics, and entry channels for MexXY are likely to be similar to other RND efflux systems (6, 7).

### Substrate selectivity by the IM transporter MexY

In the absence of MexXY structures, computational models of MexY and the full efflux system have been generated using the structure of MexAB-OprM as a guide to identify the expected functional regions for substrate recognition, such as the cleft and vestibule entry points, PBP and DBP (98–100) (Fig. 2C). However, between homologous pumps, these regions differ significantly in their amino acid composition and overall physicochemical properties (75, 76, 89, 91, 93). For example, *P. aeruginosa* MexB and MexY have overall 47% sequence identity (66% similarity) but with lower conservation in the PBP and DBP (~35% sequence identity). The resulting distinct physicochemical properties of these binding pockets in MexB and MexY likely play a crucial role in the unique substrate preferences of these transporters (99). For example, compared to MexB, the MexY DBP has fewer charged and polar residues, while the PBP is enriched in polar and charged residues. Furthermore, the combined PBP/DBP has a greater volume in MexY compared to MexB (~1,590 Å^3^ and 1,120 Å^3^, respectively). Collectively, these observations suggest that MexY is uniquely and specifically adapted through these features to accommodate larger, charged substrates, like aminoglycosides (99, 100).

Differences in the DBP not only influence substrate discrimination but also EPI affinities. For example, the broad-spectrum pyridopyrimidine EPI ABI-PP is active against both *E. coli* AcrB and *P. aeruginosa* MexB but does not inhibit *P. aeruginosa* MexY (94). The crystal structure of ABI-PP bound to AcrB revealed a binding preference for a region in the DBP that is enriched in aromatic residues (termed the “hydrophobic trap”), particularly through π–π interactions with AcrB and MexB residue F178 (94). Through homology modeling and mutational analyses, the corresponding residue in MexY, W177, was proposed to prevent inhibitor binding through steric disruptions (94).

The basis for substrate discrimination between MexY and MexB was further explored by computationally investigating differences in predicted binding affinities for substrates and nonsubstrates in the DBP regions of the two transporters (100). Contrary to initial expectations, substrates uniformly had lower predicted binding affinities for their respective transporter as compared to nonsubstrates, e.g., MexB affinities for aminoglycosides > β-lactams, and conversely in MexY, β-lactams > aminoglycosides (100). These results led to the proposal of a “Goldilocks binding affinity” hypothesis for substrate selection and efflux: substrates must bind tight enough for uptake but not so tight as to impede translocation through the pump. *In silico* homology swapping of specific residues in MexB to the corresponding amino acid of MexY could reverse the pattern of docking scores, suggesting these residues might be important for substrate recognition in MexY (100). However, further investigation into important residues predicted via computational studies, in addition to complementary biochemical and structural studies will be required for a more complete understanding of substrate selection in these efflux systems.

### MexXY can partner with multiple OMPs

Since the late 1990s, the debate regarding which OMP associates with MexXY has been controversial as the *mexXY* operon does not encode an OMP in the majority of *Pseudomonas* strains. However, the experimentally validated OMP to form a functional efflux complex with MexXY is OprM, which is encoded on the *mexAB-oprM* operon (21, 23, 101–103) (Fig. 1B). Although OprM is shared between MexAB and MexXY, OMP availability does not appear to be a limiting factor for efflux pump activity even when MexXY is overexpressed (102). As OprM is essential for MexXY function in the majority of *P. aeruginosa* strains (104), this OMP has become widely accepted as the preferred MexXY partner for multidrug resistance.

To identify whether *oprM* expression correlated with *mexXY*, the abundance of OprM was assessed in pan-aminoglycoside resistant CF isolates, but OprM expression was neither detectable nor increased compared to wild-type PAO1 (13, 105). These data led to the hypothesis that MexXY may bind an additional OMP, an idea further corroborated when a multidrug resistant isolate from Argentina, *P. aeruginosa* PA7 was sequenced and found to encode OprA within the *mexXY* operon (103, 106), i.e., *mexXY-oprA* (Fig. 1B). Furthermore, a small fragment of *oprA* was later identified downstream of *mexY* in some PAO1 and PAK genomes suggesting that they may also have once possessed *oprA,* but that it has since been lost and OprM adopted as the main MexY-associated OMP (103). Other studies have also revealed the potential for alternative OMPs with high homology to OprM to contribute to changes in MexXY activity and aminoglycoside resistance (104, 107).

Examining OMPs from other known RND systems including OpmD (encoded with MexGHI), OpmE (encoded with MexPQ), and OpmB (encoded with MuxABC) revealed that complementation of OpmB in a PAO1 (MexXY^+^ OprM^−^) background resulted in increased MICs compared to the parent strain for multiple MexXY substrates, e.g., aminoglycosides, macrolides, tetracycline, and ciprofloxacin (104). This finding suggests that OpmB, but not OpmD or OpmE, may be an additional OMP partner for MexXY. A second study identified three additional putative OMPs that contribute to aminoglycoside resistance, but are not associated with characterized RND efflux pumps, including OpmG, OpmH, and OpmI (107). Complementation of OpmG or OpmH in a *P. aeruginosa* strain PAK ∆*opmG/opmH* or PAO6609 ∆*oprM* background resulted in partial or full recovery to wild-type PAK MIC levels for the aminoglycosides tested (107); however, these results were not reproduced in a separate study (108). Recently, OpmG was found to be a member of the Major Facilitator Superfamily efflux operon, *mfsCD-opmG*, and showed no direct effect on aminoglycoside resistance in a major efflux knockout strain, *P. aeruginosa* P∆6 (PAO1∆*mexAB-oprM, mexCD-oprJ, mexEF-oprN, mexJKL, mexXY, triABC*) (109), validating the findings of the latter study (108). These conflicting data highlight potential disadvantages in exclusively relying only on cell-based studies, and such protein-protein interactions should be further investigated using complementary biochemical or structural approaches.

**Fig 3:**
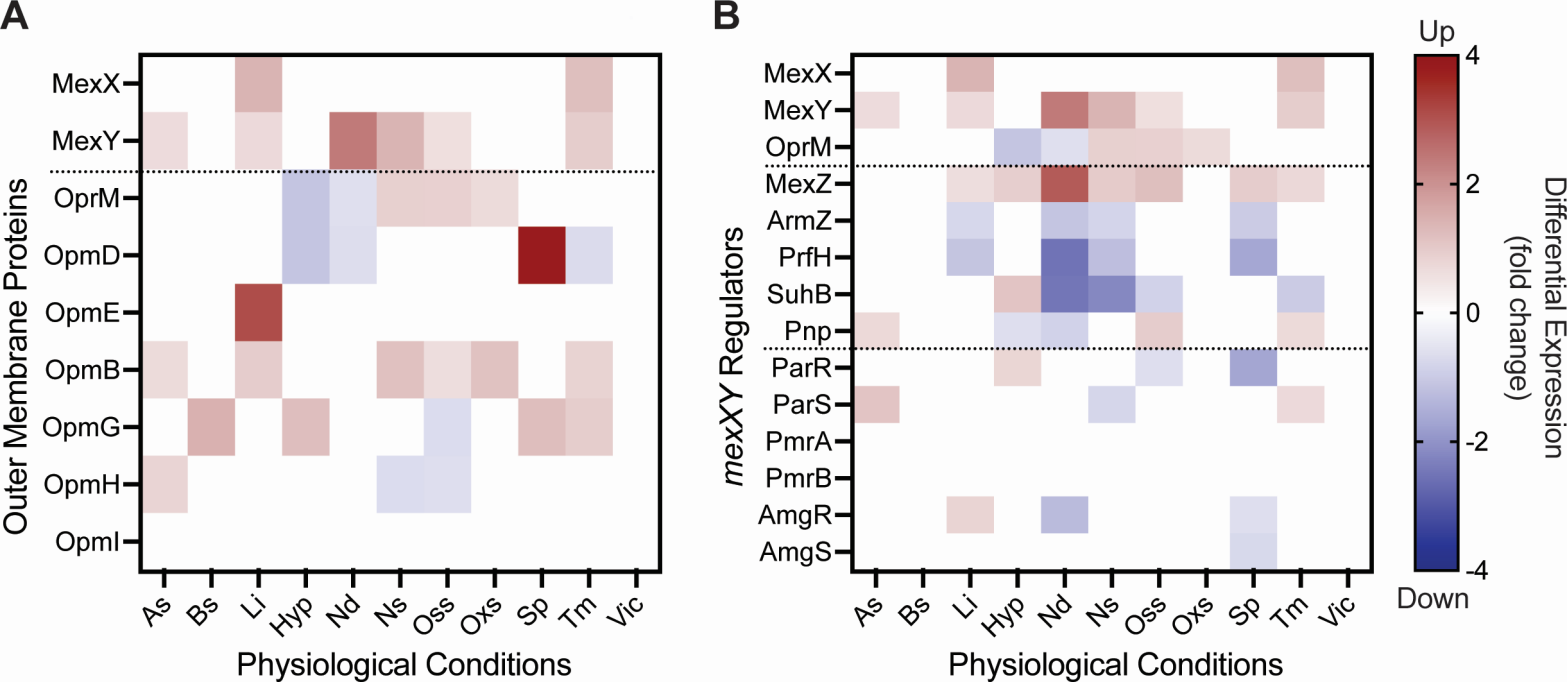
Expression of MexXY, OMPs, and regulatory proteins under different physiological conditions. Heat maps of differential gene expression data from PATHOgenex (110) for *mexX*, *mexY*, and (A) OMPs predicted to interact with MexXY, and (B) regulators predicted to either increase or decrease with MexXY expression in *P. aeruginosa* PAO1. Dotted lines separate pump components MexX/MexY (and OprM in panel B) from other proteins, and the AgrZ/AgrW1 and AgrW2 classes in panel B. The scale bar denotes color-coded increase (red) or decrease (blue) in transcript levels compared to *P. aeruginosa* PAO1 grown in LB. Distinct physiological conditions are acidic stress (As; pH 3–5), bile stress (Bs; 0.5% bile sals), low iron (Li; 250 µM 2,2-dipyridryl), hypoxia (Hyp), nutritional downshift (Nd; 1× M9 salts), nitrosative stress (Ns; 250 µM spermine NONOate), osmotic stress (Oss; 0.5 M NaCl), oxidative stress (Oxs; 0.5–10 mM H_2_O_2_), stationary phase (Sp), temperature (Tm; 41°C), or a virulence inducing condition (Vic; variable).

Further insight into potential alternative partners, in addition to OprM, for MexXY can also be gleaned using PATHOgenex (110), a public database for pathogens and their respective gene expression under eleven different physiological conditions (Fig. 3). MexX and/or MexY were found to be upregulated with multiple OMPs—OpmB, OpmE, OprM, and OpmG—under various physiological conditions, with the strongest correlation being between MexY and OpmB, further supporting their previously proposed functional association (104). Specifically, both MexY and OpmB are upregulated under acidic stress, low iron availability, nitrosative and oxidative stress, and increased temperature (Fig. 3A). As MexXY has been increasingly characterized for activities beyond multidrug efflux (discussed further below), it may be that MexXY favors alternative OMPs under distinct, nonantibiotic-induced stress conditions. However, previous studies have only considered gene deletions and resultant MIC values, making it unclear whether these changes are contributed directly by OMP binding to MexXY. Detailed biochemical and/or structural studies investigating the interactions of putative partner OMPs with MexXY are therefore needed to determine whether any of the proposed OMPs that correlate with resistance recovery in antimicrobial susceptibility testing assays can directly bind with MexXY.

## ROLE OF MexXY IN CLINICAL MULTIDRUG RESISTANCE, CELL PHYSIOLOGY, AND STRESS RESPONSES

Before the discovery of MexXY, aminoglycoside resistance in *P. aeruginosa* that was not mediated by aminoglycoside-modifying enzymes was referred to as “aminoglycoside impermeability-type resistance” (13). Since then, MexXY has been recognized to play a significant role in intrinsic resistance to all classes of aminoglycosides, including the most recent 5-deoxy-5-episubstituted aminoglycosides (111), as well as tetracycline, erythromycin, fluoroquinolones, macrolides, chloramphenicol, some cephems, trimethoprim, and carbapenem (10, 23, 101, 112–114).

Studies which identified these classes of antibiotics as substrates, either through the removal or overexpression of MexXY, further showed that some classes of substrates are preferred over others. For example, Masuda et al. (10) investigated preferred substrates for MexXY-OprM, MexAB-OprM, and MexCD-OprJ by identifying a strain where one efflux system was overexpressed (e.g., MexXY-OprM) and then subsequently deleting the remaining two pumps in this same strain (i.e., *mexAB-oprM* and *mexCD-oprJ*). Additionally, subsequent deletion of the overexpressed pump allowed comparison of MICs attributed to the overexpressed system in the absence of contributions from the other two systems (summarized in Table 2) (10). They found that while some antibiotic classes are substrates of all three RND pumps (e.g., tetracyclines), others are preferred by each individual pump: e.g., aminoglycosides for MexXY-OprM, β-lactams for MexAB-OprM, and macrolides for MexCD-OprJ. Furthermore, within each antibiotic class, a wide range of fold changes in MICs can be observed (e.g., cephems in MexXY-OprM with a range of 2- to 33-fold) depending on the specific antibiotic.

**TABLE 2:** Comparison of antibiotic MIC measurements to determine efflux profiles for MexXY-OprM and other *P. aeruginosa* RND systems (from Masuda et al. [10])^*a*^

Antibiotic	Fold change in MIC*		
	*mexXY*^+^ (*∆mexAB∆mexCD*)	*mexAB*^+^ (*∆mexCD∆mexXY*)	*mexCD*^+^ (*∆mexAB∆mexXY*)
Quinolones	16–66	31–512	32–2,048
Macrolides	8	4	128–512
Tetracycline	15–256	16–128	123–512
Aminoglycosides	32–133	1–2	0.007–1
Chloramphenicol	4	128	256
Novobiocin	1	132	8
Polymyxin B	1	1	1
Penicillins	1–8	64–3,938	1–1,024
Cephems	2–33	8–4,096	1–1,024
Oxaphems	1–2	64–512	1–8
Carbapenems	1–3	0.5–62	0.5–8

^*a*^ Antibiotic MICs (measured in μg/mL) are shown as fold-change (FC) between an overexpressing strain for each indicated RND system (+) and its isogenic triple deletion. Values are categorized using cell coloring: low (0-4 FC, white), intermediate (4-16 FC, light gray), or high impact (>16 FC, gray).

Although MexXY does not play a primary role in the removal of cephems (e.g., cefuroxime, cefpirome, cefepime, and cefozopran), MexXY-OprM can contribute to resistance by compensating for the absence of the primary resistance contributors, e.g., MexAB-OprM and β-lactamase or AmpC (21), highlighting the potential for crosstalk between RND efflux systems within the same organism. Furthermore, while *P. aeruginosa* PAO1 MexXY-OprM and PA7 MexXY-OprA share substrate profiles, only when MexXY is bound to OprA is it able to export bi-anionic β-lactams, e.g., carbenicillin and sulbenicillin (115). In addition to these well-known MexXY substrates, recent reports have suggested that MexXY may contribute to resistance to antibiotics of last resort, e.g., colistin (116) and imipenem (114, 117), either as a substrate or through downstream effects (116, 117), which has serious implications for future therapeutic interventions.

Aminoglycoside, carbapenem, and colistin resistance have been associated with increased expression of *mexXY* in clinical strains from around the world (118–126), as well as in migratory birds, felines, and cows (105, 127–129). Overexpression of multiple efflux pumps, particularly MexAB-OprM and MexXY-OprM/OprA, is common in clinical isolates and contributes to multidrug-resistant phenotypes (20, 130) either directly or indirectly, as a foundation for additional resistance mechanisms to emerge (37, 55, 131, 132). Of particular relevance for individuals with CF, MexXY overexpression is a major contributor to aminoglycoside resistance (13, 105). Additionally, the overexpression of efflux pumps, like MexXY, can provide a competitive advantage through increased fitness in the presence of antibiotics with no decrease in fitness compared to wild-type strains in the absence of antibiotics (102). Poor clinical outcomes for *P. aeruginosa* infections are often due to a combination of virulence factors such as type III secretion systems and multi-drug resistance, e.g., via overexpression of efflux pumps (48, 133). To address the high prevalence of overexpression of MexXY in clinical isolates, an advancement in therapeutic strategies is desperately needed, such as the development of EPIs.

Overexpression of MexXY is also implicated in physiological functions beyond the efflux of antibiotics. For example, MexXY has been associated with responses to the consequences of ribosome dysregulation, possibly through export of truncated, or aberrant peptides (33, 35–37, 56), and responding to membrane stress by export of lipopolysaccharide components (116, 134, 135). Furthermore, a strain overexpressing MexXY showed a significant increase in expression of nitrate respiratory pathway genes (e.g., *IsnirQ, norB, nosZ, fhp*) that produced and consumed the greatest amounts of nitric oxide as compared to strains overexpressing other RND systems (136). These findings suggest that overexpression of efflux pumps, including MexXY, can rewire physiological pathways within the cell to counteract fitness costs associated with resistance.

Analysis of PATHOgenex (110) data also reveals a correlation between the upregulation of MexXY and the regulators associated with MexZ (i.e., MexZ, ArmZ, PrfH, SuhB, and PNPase) and TCS (i.e., ParRS, PmrAB, and AmgRS) (Fig. 3B). Overall, overexpression of MexZ closely resembled expression of MexXY in low iron, nutritional deficiency, nitrogen and oxidative stress, and temperature. MexZ is a repressor for the *mexXY* operon, therefore, overexpression of MexZ should result in repression of *mexXY*, as seen in hypoxia or stationary phase. The upregulation of MexXY in these conditions must therefore be due to a different regulator. Interestingly, as SuhB is a known repressor of ArmZ (35), SuhB downregulation when MexZ is upregulated provides a potential alternative pathway for MexZ derepression. Two-component systems which accumulated mutations (e.g., ParRS, PmrAB, and AmgRS) were also shown to increase MexXY expression. Therefore, we additionally anticipate that these systems may be downregulated when MexXY is upregulated, and this is indeed observed in ParR (oxidative stress), ParS (nitrogen stress), and AmgR (nutritional deficiency). However, the opposite is true for ParS (acidic stress and temperature) and AmgR (low iron) as these regulators were overexpressed in these conditions, while PmrAB was not found to be differentially expressed in any of the tested conditions. While these data are correlative, they provide initial insights into potential physiological conditions under which MexXY is “turned on” and the conditions relevant for specific regulators. These insights can guide future experimental studies to determine functional causation between preferred pathways and distinct environmental conditions.

## DEVELOPMENT OF *MexXY* EPIS

### Broad spectrum EPIs

The first two broad-spectrum EPIs that showed promise against RND transporters were phenylalanine arginyl β-napthylamide (PAβN or MC-207,110) (137) and carbonyl cyanide-m-chlorophenylhydrazone (CCCP) (138, 139). PAβN is a cationic dipeptide that binds in the hydrophobic trap within the DBP of the MexY transporter protein where it blocks the peristaltic movement of the pump (140). PAβN inhibits multiple Mex RND systems (141), but is not clinically useful due to acute toxicity (142) and because it increases resistance to aminoglycosides when used in combination with these drugs (72). CCCP suppresses the electron transport chain resulting in a decrease in the proton transport that is required for pump function (138, 139). While CCCP is commonly used in the lab to study efflux pump activity, clinical application of this compound is not possible due to its extreme toxicity to mammalian cells while also increasing resistance to antibiotics by rendering bacterial cells metabolically inactive (138, 139). These unfortunate side effects of PAβN and CCCP highlight at least some of the potential downfalls with broad-spectrum EPIs.

### EPIs active against *Pseudomonas* spp.

While significant effort has been expended in the advancement of EPIs for the *Enterobacteriaceae* family and *Staphylococcus aureus*, corresponding development for *Pseudomonas* spp. is currently far behind. Challenges to EPI discovery in *Pseudomonas* include decreased permeability of the outer membrane and increased abundance of RND efflux systems as compared to *E. coli* (143). Despite these limitations, a few compounds are able to cross the *Pseudomonas* outer membrane and inhibit Mex efflux systems, including plant-derived natural compounds (e.g., berberine and curcumin), microbe-derived compounds (e.g., 3,4-dibromopyrrole-2,5-dione), arylpiperazines (e.g., NMP), pyridopyrimidine (e.g., D13-9001), and TXA compounds (e.g., TXA09155) (143). As the efficacy, synthesis, and limitations to each of these groups of compounds as EPIs have been previously reviewed in depth (144, 145), here we focus on promising MexXY-OprM-specific inhibitors.

### *MexXY specific EPI*s

Despite its relatively low efficacy, one of the currently most promising inhibitors of MexXY function is berberine, a natural alkaloid extracted from plants such as *Hydrastis canadensis* and *Berberis aristate* (146, 147). Since the first discovery of berberine’s weak EPI activity (147), three studies have shown promising improvements in its activity as a potential inhibitor. First, Su et al. showed that concurrent administration of berberine with imipenem resulted in synergistic inhibition of efflux by MexXY due to increased berberine uptake via imipenem-induced membrane damage (117). Second, Kotani et al. synthesized 11 berberine derivatives and showed that 13-(2-methylbenzyl) berberine (“13-o-MBB”) has potent activity against MexXY (148). This derivative reduced aminoglycoside MICs by 4- to 128-fold in *P. aeruginosa*, while also having activity against *Achromobacter xylosoxidans* and *Burkholderia cepacia* (148). However, this EPI showed cytotoxicity against a human epithelial colorectal adenocarcinoma cell line at the concentration (256 µg/mL) necessary for pump inhibition (148), and will therefore not proceed to animal studies. Third, Kavanaugh and Mahoney et al. (149) identified a di-berberine conjugate with a propane linker (Ber-C3) that was effective at reducing resistance to substrates (e.g., aminoglycosides and tigecycline) by 2- to 16-fold in *P. aeruginosa* PAO1, PA7, PA14, and pan-aminoglycoside resistant clinical isolates. Promisingly, Ber-C3 was found to be nontoxic to red blood cells at the concentrations needed for efflux inhibition. The molecular mechanism of action of berberine and its derivatives against MexY remains to be determined, and future high-resolution structural insights would be invaluable in accelerating development of new derivatives with enhanced activity.

### Future considerations for EPI development

One EPI (MP-601,205 or pentamidine) was the first of its class to enter into Phase 1 clinical trials in 2007 for the treatment of infections in CF patients and for ventilator-associated pneumonia. However, trials were discontinued due to issues with membrane perturbation, and the true mechanism of action was shown to be DNA replication (142, 150). Although this specific EPI did not make it to market, other pentamidine analogs as nontoxic adjuvants have been discovered and show promise against additional Gram-negative bacteria (151). This scenario highlights the importance of considering combination antibiotic-adjuvant therapies in the clinical landscape for overcoming multidrug resistance. A second combination therapy, conjugate antibiotics, e.g., meropenem fused to an aminoglycoside, as potential efflux pump inhibitors have also come into focus as potential therapeutics (135). However, when using combination antibiotics to inhibit efflux pump action, caution will need to be taken because one of the antibiotics may increase expression of the pump, even though it is not a substrate (152). Finally, a potential alternative therapeutic strategy is the use of *P. aeruginosa* bacteriophage OMKO1 for phage therapy (153). OMKO1 recognizes OprM for entry, and to avoid infection, OprM is downregulated, resulting in increased susceptibility to antibiotics and providing protection against all efflux systems which use OprM as an OMP in *P. aeruginosa*.

## CONCLUSIONS AND FUTURE PERSPECTIVES

The *P. aeruginosa* MexXY-OprM efflux pump is an important contributor to both acquisition of multidrug resistance and other key facets of *Pseudomonas* physiology. Importantly, as the *mexXY* operon is not constitutively expressed, numerous complex and interconnected pathways have emerged that appear to contribute to the regulation of *mexXY* expression. The critical role of ArmZ in controlling *mexXY* overexpression and resulting multidrug resistance is now well established. However, newly identified components, such as SuhB and PNPase, and an ArmZ-independent regulatory pathway have revealed the increasing complexity involved and underscore how much remains to be understood at a molecular level. Additionally, the mechanism of regulation via a leader peptide of an anti-repressor protein appears to be unique to MexXY, highlighting how such distinctive regulation under antibiotic-stress conditions might significantly influence the clinical relevance of the MexXY-OprM efflux pump. A deeper understanding of MexXY’s regulation through these varied mechanisms could reveal new targets by which to restore the efficacy of existing antibiotics and combat resistance in *P. aeruginosa*.

As deep structural insight into the activity of MexXY is currently lacking, and as a result, detailed analyses of specific mechanistic features of this system are more limited than for other RND pumps. Such structural insights will undoubtedly become available soon and should deepen our understanding of MexXY substrate specificity (e.g., its unique capacity in *P. aeruginosa* to efflux aminoglycosides) and enable structure-guided improvements in ongoing EPI development efforts. Among the future challenges that will need to be addressed are increasing EPI efficacy while also overcoming the solubility and toxicity issues often observed for current MexXY-specific EPIs. Together, new structural and mechanistic insight into MexXY action, coupled with unraveling the complex mechanisms of *mexXY* regulation, putative additional OMP binding proteins, and the system’s broader contributions to *Pseudomonas* physiology will set the scene for developing MexXY-targeted therapeutics, and as a consequence, may establish new strategies that will be more broadly applied to other Gram-negative pathogens of significant concern.

## ADDENDUM

A cryoEM structure of MexY at modest resolution (PDB 9E9F) was reported while this minireview was in production.
